# Accuracy Analysis of SINS/CNS Integrated Attitude Determination Based on Simplified Spatio-Temporal Model

**DOI:** 10.3390/s25226898

**Published:** 2025-11-12

**Authors:** Conghai Ruan, Hanxu Li, Chonghui Li, Shaojie Chen, Zhiqiang Hong

**Affiliations:** College of Geospatial Information, Information Engineering University, Zhengzhou 450001, China

**Keywords:** CNS/INS, simplified spatio-temporal model, attitude determination, calibration, coordinate system

## Abstract

For ground-based Celestial Navigation System/Strapdown Inertial Navigation System (CNS/SINS) integrated navigation with arcsecond-level accuracy, the current spatio-temporal transformation model involves a considerable amount of astronomical knowledge, making it difficult for ordinary navigation professionals to quickly master and operate. There has been no strict argumentation on which parameters can be simplified in the calculation process. Under the premise of ensuring that the attitude accuracy of ground integrated navigation meets the requirement of 5 arcseconds, through argumentation and quantitative analysis, the complex links in the spatio-temporal transformation model that contribute minimally to the final attitude measurement accuracy can be eliminated, significantly reducing the complexity of the model and lowering the threshold for its use. The factors considered in this paper include proper motion, annual parallax, light deflection, aberration of light, details of the precession-nutation model, details of the time system, and calibration parameters. Factors contributing less than 0.1 arcsecond to the accuracy during the coordinate transformation process are ignored or approximately simplified. Error analysis shows that the corrections for annual parallax and light deflection have negligible effects on accuracy. Except for the calculation of the Earth’s rotation angle, which requires a relatively precise UT1-UTC time, the time input in the calculation process of other astronomical parameters can directly use UTC time. Experimental measurements show that the calibration parameters obtained by the method in this paper have high robustness, and the parameter accuracy meets the requirements of attitude calculation. The proposed simplified spatio-temporal model reduces the computational load by 90%, can meet the arcsecond-level attitude measurement accuracy requirements of ground-based CNS/INS integrated navigation, and has the potential to be extended to more general dynamic or air/space-based intelligent navigation scenarios.

## 1. Introduction

In complex ground environments, the denial, interference, and spoofing of Global Navigation Satellite System (GNSS) signals are the fatal bottlenecks that restrict navigation accuracy [1]. The SINS has the characteristic of full autonomy, but its attitude measurement error accumulates over time and cannot meet the requirements of long-duration high-precision attitude determination [2]. The CNS provides an absolute attitude reference by observing the star vector, and its error does not drift over time, theoretically achieving arcsecond-level accuracy [3]. However, single celestial navigation is limited by weather conditions and observation frequency, making it difficult to meet the real-time requirements of high-dynamic carriers [4]. The SINS/CNS can achieve complementary advantages. With its strong autonomy and the characteristic that attitude error does not accumulate over time, it has become a core technology for high-precision attitude determination and plays an irreplaceable role in spacecraft orbit determination, long-duration flight vehicle navigation, and other fields [5].

In the SINS/CNS combination, CNS not only provides precise attitude calibration but also assists INS in estimating the drift of gyroscopes, thereby enhancing the recursive accuracy of SINS [6]. Traditionally, it is believed that celestial navigation is more suitable for aviation/astronautic platforms with high sky visibility, while its ground applications are often overlooked. Recent studies have shown that under static/quasi-static ground conditions, the attitude determination accuracy of SINS/CNS can reach 3–5 arcseconds, approaching the limit of optical measurement [7,8]. Cheng (2019) used a one-thousandth inertial navigation system combined with a high-precision star sensor to calculate the carrier’s attitude, achieving an attitude accuracy of less than 3” in 3.5 h of static conditions [9]. Li et al. (2024) proposed a robust SINS/CNS combination filtering algorithm, significantly improving the attitude determination accuracy of ground carriers, with less than 3” in the horizontal direction and less than 10” in the heading direction [10].

During the SINS/CNS integration process, the recursive position, velocity and attitude results of SINS are generally in the Earth coordinate system, while CNS calculates the position and attitude by observing celestial body signals, with the reference frame being the celestial sphere. Take the Hipparcos star catalog as an example, it uses the J2000 coordinate system as the reference benchmark [11]. To achieve the fusion of the two types of navigation information, the transformation of the spatio-temporal reference model is required first. The current model is rather complex and requires strict handling of more than ten astronomical effects such as precession, nutation, Earth rotation parameters, stellar proper motion, annual parallax, and aberration of light, involving multiple reference frame conversions from International Celestial Reference System (ICRS) to International Terrestrial Reference System (ITRS), which demands a profound background in astronomy from the user [12]. Focusing on the ground arcsecond-level integrated attitude determination accuracy, some effects in the existing model (such as parallax, diurnal aberration of light, and light deflection, etc.) have an impact level far below 1” in short-term ground attitude determination, but significantly increase the system complexity and computational burden [13]. Although some studies have proposed to ignore the influence of some effects, the current simplified models are mostly based on experience and have not strictly demonstrated under what scenarios which effects can be ignored. Yan et al. (2022) discussed the impact of the simplified time model on SINS/CNS attitude determination, ignored and approximately simplified factors less than 0.1 arcsecond in the coordinate transformation process, and verified through simulation experiments that the accuracy of the simplified time model meets the requirements of astronomical sensor attitude measurement, but lack of measured data to further verify the validity of the model [14].

To achieve a more stable and accurate attitude, during the conversion process from pixel coordinates to camera coordinates, high-precision calibration of camera parameters is required. Among them, camera distortion is a factor that must be given priority consideration. However, for the arcsecond-level ground combined orientation requirements, the specific orders that need to be taken into account in the radial and tangential distortions of the camera have not yet formed strict theoretical arguments. The internal structure and parameters of star sensors may undergo slight changes due to the vibration or temperature variations in the ground working environment. Regular and timely calibration of star sensor parameters is a prerequisite for achieving high-precision attitude determination. Traditional calibration methods such as the Direct Linear Transformation (DLT) method and Zhang’s calibration method can achieve high accuracy in camera parameter calibration [15,16]. Especially when the application requires high precision and the camera’s internal parameters do not change frequently [17]. However, they increase the calibration cost and are not applicable in situations where calibration objects cannot be used. Calibration methods based on active vision do not require calibration objects but require the camera to perform special movements such as rotation or translation [18,19]. Through the particularity of the movement, the internal orientation elements of the camera can be calculated. This method is not suitable for situations where the camera’s movement is unknown or uncontrollable. Self-calibration methods estimate the internal orientation elements of the camera based on the correspondence of image points between images, only using the constraints inherent in the camera’s internal parameters. They are highly flexible but have relatively low calibration accuracy and strong scene dependence [20,21,22].

Based on the above research, this paper starts from the perspective of reducing the complexity and usage threshold of the spatio-temporal model. Under the premise of ensuring attitude accuracy in specific application scenarios, through demonstration and quantitative analysis, it eliminates the complex links in the spatio-temporal transformation model that contribute minimally to the final accuracy. In the process of converting pixel coordinates to camera coordinates, a calibration method that uses natural “zero-cost” stars as high-precision benchmarks is adopted to simplify the calibration model, reduce the parameter deviation between the laboratory and the actual environment, and achieve an “immediate inspection and immediate use” effect. Finally, the effectiveness of the proposed method is verified through actual measurement experiments.

## 2. Construction of a Simplified Spatio-Temporal Model

This section first introduces what corrections need to be made during the classic International Celestial Reference System (ICRS) transformation to the navigation coordinate system and the time systems used; then it approximates, simplifies and omits the above corrections and analyzes their errors; finally, it presents the star method calibration model adopted for the transformation from pixel coordinates to camera coordinates. In the CNS/INS integrated navigation system, the improvement of overall pose accuracy mainly relies on the precision enhancement of the CNS. Therefore, this article does not elaborate on the implementation details of the integrated navigation algorithm.

### 2.1. Conversion from ICRS to Navigation Coordinate System

**Figure 1:**
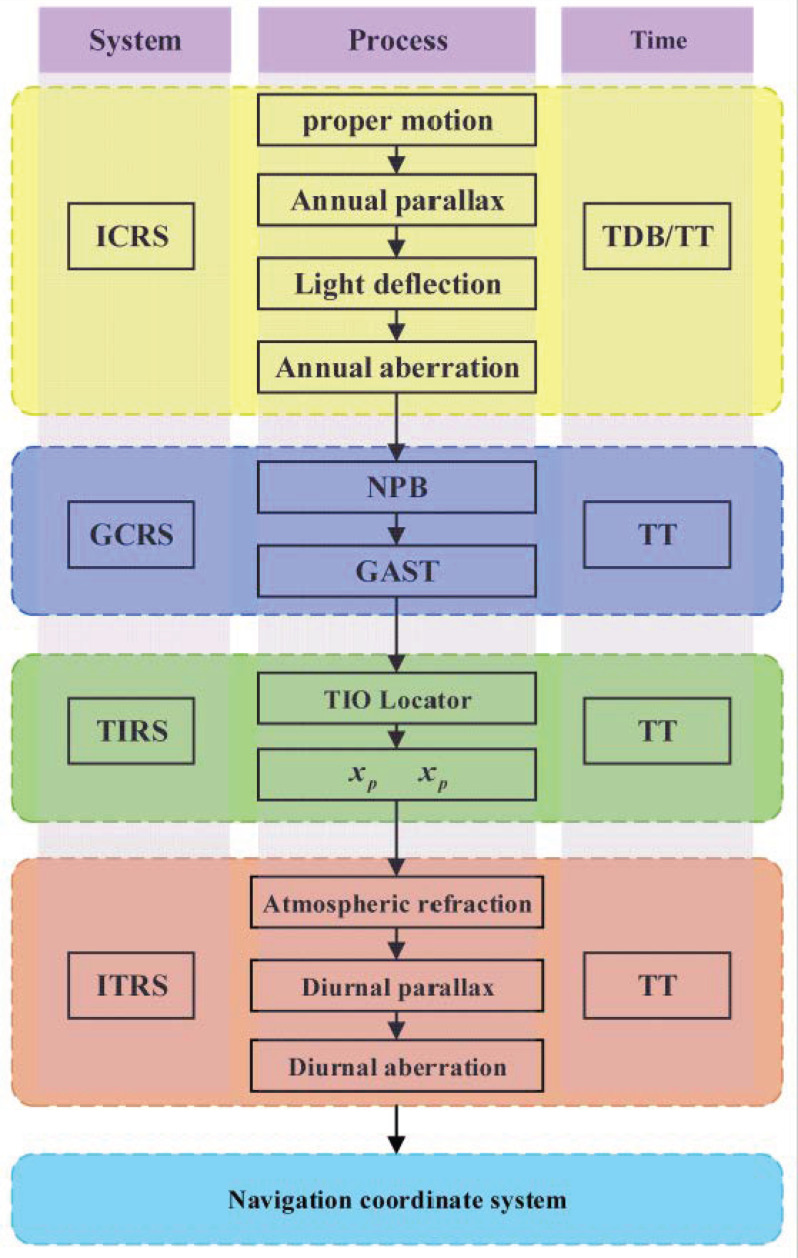
The conversion process from ICRS to navigation coordinate system.

Figure 1 shows the conversion process from ICRS to Navigation Coordinate System. The star point coordinates need to be corrected for proper motion, light deflection, annual parallax and annual aberration from ICRS to Geocentric Celestial Reference System (GCRS), and the time system used is Barycentric Dynamical Time (TDB). After correction of precession, nutation and Greenwich Apparent Sidereal Time (GAST), it can be transferred to the coordinates of the Terrestrial Intermediate Reference System (TIRS). After correction of polar shift, it can be transferred to the International Terrestrial Reference System (ITRS). After corrections for diurnal parallax, diurnal aberration and atmospheric refraction, it can be transferred to the navigation coordinate system, with the reference time being Terrestrial Time (TT) [12].

The time systems involved in the conversion from ICRS to ITRS are TDB, TT, International Atomic Time (TAI), Coordinated Universal Time (UTC) and Universal Time (UT1). The main difference between TT and TDB is caused by the relativistic effect of the change in the gravitational potential of the Earth-Moon system’s motion around the Sun. There is no long-term trend term between them, and the maximum difference is only about 1.7 ms, which can be ignored in the calculation [23]. Each time system can be mutually transformed by Equation (1):

(1) $$\begin{array}{cc} \mathrm{UT}1\; & =\;\mathrm{UTC}\;+\;\left(\mathrm{UT}1\;-\;\mathrm{UTC}\right)\\ \mathrm{TAI}\; & =\;\mathrm{UTC}\;+\;\mathrm{DAT}\\ \mathrm{TT}\; & =\;\mathrm{TAI}\;+\;32.184\;s\\ \mathrm{TDB} & \approx \mathrm{TT} \end{array}$$

In Equation (1), DAT represents the leap second of UTC, currently taking a value of 37. The specific values of UT1-UTC can be found on the official website of the International Earth Rotation Service (IERS) [24].

The Julian Day (JD) is a long-term and continuous method of counting days that does not involve concepts such as years and months, and is widely used in astronomy. The calculation formula of the Julian day is [25]:

(2) $$\mathrm{JD}=1721013.5+367\times Y-\mathrm{int}\{[\frac{7}{4}[Y+\mathrm{int}(\frac{M+9}{12})]]\}+\mathrm{int}(\frac{275\times M}{12})+D+\frac{h}{24}$$

In Equation (2), the constant 1,721,013.5 is the JD at 0:00 on 1 January, AD. Y, M, and D represent the year, month, and day in the Gregorian calendar, respectively, and h represents the hour.

The Julian Century is a commonly used unit of time with long intervals in astronomy. Given the UTC moment, the number of centuries away from J2000 can be obtained, that is:

(3) $$T=(\mathrm{JD}-{\mathrm{JD}}_{0})/36525$$

The corresponding JD in Equation (3) is 2,451,545.0 days.

Star point extraction and identification are carried out based on the star map captured by the star sensor. The right ascension and declination of the star are determined by the star catalog. The star catalog adopted in this paper is the Hippargo catalog, which takes ICRS as the reference frame and has an epoch of 2000.0. The position, speed and attitude parameters of the integrated navigation we obtain are generally in the Earth coordinate system, so the observation position is determined in the ITRS coordinate system. Other positions are intermediate conversion results. The conversion of these coordinate systems not only includes three-dimensional spatial coordinates but also requires an additional time coordinate to form event coordinates to express the event at a certain point in time. The common method to achieve the transformation between coordinate systems is the Euler angle rotation method.

### 2.2. Spatial Model Simplification

In astronomical navigation, it is necessary to calculate the right ascension and declination of celestial bodies at the moment of observation. The Hippargo star catalog includes the mean position, proper motion, and annual parallax of stars in the standard epoch J2000.0. Based on this information, the instantaneous position of stars can be calculated. Ignore correction items with an impact of less than 0.1 arcseconds, the calculation process is as follows [14,26,27]:

1. Calculate the JD corresponding to the TDB at the moment of observation

The time recorded at the moment of astronomical navigation and positioning observation is UTC time. According to Equations (1) and (2), it can be converted into TT and JD at the moment of observation.

2. Calculate the heliocentric position $r_{1}$ of the J2000.0 epoch star

The heliocentric position serves as a bridge connecting the inherent position of a star and the observational deviation caused by the Earth’s revolution. To eliminate the influence of the Earth’s revolution on observations, it is necessary to shift the mean position of the star J2000.0 in the star catalog to the heliocentric position.

(4) $$r_{1}=d\cdot \left[\begin{array}{c} \cos\delta_{0}\cos\alpha_{0}\\ \cos\delta_{0}\sin\alpha_{0}\\ \sin\delta_{0} \end{array}\right]$$

In Equation (4), $\alpha_{0}$ and $\delta_{0}$ are the mean position of the star catalog in the J2000.0 epoch, and *d* is the heliocentric distances of the star. Calculate according to Equation (5):

(5) $$d=206264.8062470944^{\prime \prime }/\pi$$

In Equation (5), π represents the annual parallax of the star. When π is unknown or 0, $\pi =10^{-7}$ is taken for calculation.

3. Calculate the heliocentric position $r_{2}$ after proper motion correction

Stars are not fixed on the celestial sphere but have long-term motion, and their positions change over time. The calculation formula for the proper motion correction of stars is:

(6) $$v=\left[\begin{array}{l} v_{x}\\ v_{y}\\ v_{z} \end{array}\right]=\left[\begin{array}{c} 15\mu_{\alpha }\cos\delta_{0}/\rho \\ \mu_{\delta }/\rho \\ V\cdot k\cdot \pi /\rho \end{array}\right]$$

In Equation (6), ρ=206264.8062470964 and k=21.094953. Equation (7) represents the proper motion of a star in space every hundred years. According to Equation (5), it can be transformed into the basic coordinate system $v_{0}$ of the star catalog:

(7) $$v_{0}=R_{z}\left[-(90+\alpha_{0})\right]R_{x}\left[-(90-\delta_{0})\right]v$$

Then the corrected heliocentric position $r_{2}$ after proper motion correction is:

(8) $$r_{2}=r_{1}+(t-t_{0})v_{0}$$

In Equation (8), t represents the observed epoch, and $t_{0}$ represents the observed epoch for compiling the star catalog. It can be seen from this that the proper correction of a star accumulates over time. 25 years have passed since the J2000.0 epoch. If the proper correction is ignored, the apparent position calculated based on the epoch’s mean position will deviate from the actual observed results.

4. Calculate the geocentric position $r_{3}$ after the annual parallax correction

The Earth’s revolution around the Sun causes the observer’s spatial position to change with the seasons, resulting in a slight periodic shift in the direction of star observation, which is known as annual parallax. The most important requirement for annual parallax correction is the radial path of the solar system’s center of mass beyond the center of the earth. However, the coordinate of the solar system’s center of mass at the center of the earth is not a fixed value; it is a dynamic value that changes rapidly over time. Let the position vector of the solar system’s center of mass coordinate at the center of the earth be $r_{e}$, then the expression of $r_{3}$ is:

(9) $$r_{3}=r_{2}-\pi r_{e}$$

In navigation, we usually care whether the maximum angular displacement caused by parallax reaches an observable order of magnitude. For annual parallax, since the distance from the heliocentric center of the vast majority of stars is much greater than the distance from the heliocentric center to the earth’s center, it can generally be ignored. Only a very small number of stars have an annual parallax greater than 0.01″. Therefore, in ground CNS/INS integrated navigation, the correction of annual parallax does not need to be carried out.

5. Calculate the geocentric position $r_{4}$ after the light deflection correction

Before the light emitted by stars reaches the Earth, if it passes through the gravitational field of a massive celestial body, its propagation path will bend. $r_{4}$ can be calculated according to the following formula:

(10) $$r_{4}=r_{3}-\frac{2GM}{c^{2}}\cdot \frac{D}{D^{2}}\left[\frac{1}{r_{e}}(r_{e}\cdot r_{3})+(r_{e}-r_{2})\right]$$

In Equation (10), $D=r_{e}-(r_{e}\cdot r_{3})\cdot r_{3}/r_{3}^{2}$, $\frac{2GM}{c^{2}}={1.9741257\times 10}^{-8}AU$. *G* is the gravitational constant, and M is the mass of the sun. When observations are very close to the sun, a maximum deviation of 1.75″ can be caused. However, for ground-based CNS/INS integrated navigation systems, solar observations are usually avoided, so the light deflection does not need to be corrected.

6. Calculate the geocentric position $r_{5}$ after the annual aberration correction

Aberration is a phenomenon of visual direction deviation caused by the relative motion between the observer and the light source. The annual aberration has the same magnitude of impact on all stars. It can be calculated by Equation (11):

(11) $$r_{5}=r_{4}+\left|r_{3}\right|\cdot V_{e}/c$$

In Equation (11), $V_{e}$ represents the orbital speed of the Earth, with the unit being astronomical units per day. The speed of light in astronomical units can be expressed as c=173.144633. Suppose the orbital speed of the Earth is $V_{e}=0.017202$, then the magnitude of the aberration of light is κ≈20.49″. Therefore, regardless of the distance of the star, as long as its light is received by Earth observers, it will be affected by the annual aberration and must be uniformly corrected.

7. Calculate the corrected geocentric position $r_{6}$ after precession and nutation correction

The axis of rotation of the Earth is not fixed in inertial space but constantly oscillates. This swing will cause the Earth’s axis to move clockwise around the north ecliptic pole. In addition, the Earth’s axis itself is also making slight tremors. The movement of the former is precession, while that of the latter is nutation. These two corrections cannot be ignored. By using the IAU1976 precession model, the precession matrix can be calculated:

(12) $$P=R_{X}(-Z_{A})R_{Y}(\theta_{A})R_{Z}(-\zeta_{A})$$

In Equation (12), the equatorial precession parameter can be expressed as:

(13) $$\left\{\begin{array}{c} \zeta_{A}=2306.2181\prime \prime T_{TDB}+0.301880\prime \prime T_{TDB}^{2}+0.017998\prime \prime T_{TDB}^{3}\\ \theta_{A}=2004.3109\prime \prime T_{TDB}-0.426650\prime \prime T_{TDB}^{2}-0.041833\prime \prime T_{TDB}^{3}\\ Z_{A}=2306.2181\prime \prime T_{TDB}+1.094687\prime \prime T_{TDB}^{2}+0.018203\prime \prime T_{TDB}^{3} \end{array}\right.$$

In Equation (13), if the $T_{TDB}$ = 0.25 century number is taken, then the maximum value $1.094687\prime \prime T_{TDB}^{2}$ in its higher-order terms is approximately 0.068″. Ignoring quantities less than 0.01″, Equation (13) can be expressed as:

(14) $$\begin{array}{c} \zeta_{A}\approx 2306.2181\prime \prime T_{TDB}\\ \theta_{A}\approx 2004.3109\prime \prime T_{TDB}\\ Z_{A}\approx 2306.2181\prime \prime T_{TDB} \end{array}$$

Although the IAU2006 precession model is currently in use and its accuracy can reach the level of 1 micro-arcsecond, for ground CNS/INS integrated navigation, the accuracy of the IAU1976 precession model is sufficient.

The nutation matrix N can be calculated by using the IAU1980 nutation model:

(15) $$N=R_{X}(-\varepsilon -\Delta \varepsilon )R_{Z}(-\Delta \psi )R_{X}(\varepsilon )$$

In Equation (15), Δψ and Δε are, respectively, the nutation in longitude and the nutation in Obliquity, which can be calculated by Equation (16):

(16) $$\left\{\begin{array}{c} \Delta \psi =\sum_{i=1}^{106}(A_{i}+A_{i}^{\prime }T_{TDB})\sin\sum_{j=1}^{5}N_{jm}F_{j}\\ \Delta \varepsilon =\sum_{i=1}^{106}(B_{i}+B_{i}^{\prime }T_{TDB})\sin\sum_{j=1}^{5}N_{jm}F_{j} \end{array}\right.$$

In Equation (16), $A_{i}$, $A_{i}^{\prime }$, $B_{i}$, and $B_{i}^{\prime }$ are constants, and $N_{jm}$ is an integer, which can be obtained from coefficient table of the IAU1980 nutation series, as shown in Table 1. $F_{1}$ to $F_{5}$ represent the fundamental arguments: the Moon’s mean anomaly, the Sun’s mean anomaly, the Moon’s mean argument of latitude, the mean elongation of the Moon from the Sun, and the mean longitude of the Moon’s ascending node, respectively. Their specific expressions are as follows:

(17) $$\left\{\begin{array}{c} F_{1}=l=134{}^{\circ}57\prime 46.733\prime \prime +(477000{}^{\circ}+198{}^{\circ}52\prime 02.633\prime \prime )T_{TDB}+31.310\prime \prime T_{TDB}^{2}+0.064\prime \prime T_{TDB}^{3}\\ F_{2}=l^{\prime }=357{}^{\circ}31\prime 39.804\prime \prime +(35640{}^{\circ}+359{}^{\circ}03\prime 01.224\prime \prime )T_{TDB}-0.577\prime \prime T_{TDB}^{2}+0.012\prime \prime T_{TDB}^{3}\\ F_{3}=F=93{}^{\circ}16\prime 18.877\prime \prime +(483120{}^{\circ}+82{}^{\circ}01\prime 03.137\prime \prime )T_{TDB}-13.257\prime \prime T_{TDB}^{2}+0.011\prime \prime T_{TDB}^{3}\\ F_{4}=D=297{}^{\circ}51\prime 01.307\prime \prime +(444960{}^{\circ}+307{}^{\circ}06\prime 41.328\prime \prime )T_{TDB}-6.891\prime \prime T_{TDB}^{2}+0.019\prime \prime T_{TDB}^{3}\\ F_{5}=\Omega =125{}^{\circ}02\prime 40.28\prime \prime +(1800{}^{\circ}+134{}^{\circ}08\prime 10.539\prime \prime )T_{TDB}-7.455\prime \prime T_{TDB}^{2}+0.008\prime \prime T_{TDB}^{3} \end{array}\right.$$

In Equation (17), when $T_{TDB}$ = 0.25 centuries, only the coefficients of $A_{i}$, $A_{i}^{\prime }$, $B_{i}$, and $B_{i}^{\prime }$ values that are greater than 0.1″ are considered. At this point, the approximate expression of Equation (16) can be represented by Equation (18), and through this approximation, the computational load is approximately 90% lower than that of the rigorous model in Novas—this offers certain advantages for embedded applications.

(18) $$\left\{\begin{array}{cc} \Delta \psi \approx & A_{1}\sin(N_{51}F_{5})+A_{2}\sin(2(N_{23}F_{3}+N_{24}F_{4}+N_{25}F_{5}))+\\ & A_{3}\sin(2(N_{33}F_{3}+N_{35}F_{5}))+A_{4}\sin(2N_{45}F_{5})+A_{5}\sin(N_{52}F_{2})\\ \Delta \varepsilon \approx & B_{1}\sin(N_{51}F_{5})+B_{2}\sin(2(N_{23}F+N_{24}F_{4}+N_{25}F_{5})) \end{array}\right.$$

In summary, $r_{6}$ can be expressed as:

(19) $$r_{6}=NP\cdot r_{5}$$

8. Calculate the corrected geocentric position $r_{7}$ after Earth’s rotation correction

GAST establishes the connection between the Earth’s rotation and the celestial reference frame. The calculation formula of GAST is:

(20) GAST=GMST+Δψ⋅cosε

In Equation (20), Δψ represents the nutation in longitude, ε is the true Obliquity of the Ecliptic, and GMST is the Greenwich Mean Sidereal Time. In the IAU2000A model, GMST is expressed as:

(21) $$GMST=\theta_{E}+Ec$$

In Equation (21):

(22) $$\theta_{E}=2\pi (0.7790572732640\;+\;1.00273781191135448d\cdot JD_{UT1})$$

(23) $$Ec\approx -\;0.014506^{2}\;-\;4612.156534^{2}T_{TDB}$$

The rotation matrix of the Earth’s rotation is:

(24) $$R=R_{Z}(-GAST)$$

Then $r_{7}$ can be expressed as:

(25) $$r_{7}=R\cdot r_{6}$$

9. Calculate the geocentric position $r_{8}$ after polar shift correction

The Earth’s axis of rotation slightly oscillates within the Earth’s body, resulting in differences between the instantaneous Earth coordinate system and the protocol Earth coordinate system. The polar shift matrix W is:

(26) $$W=R_{Y}(-x_{p})R_{X}(-y_{p})R_{Z}(s^{\prime })$$

In Equation (26), $x_{p}$ and $y_{p}$ are the values of the instantaneous pole relative to the origin of the International Protocol, provided by IERS $s^{\prime }$ is the TIO Locator, which is a tiny value dependent on time and can be ignored in the calculation. Then $r_{8}$ can be expressed as:

(27) $$r_{8}=W\cdot r_{7}$$

10. Calculate the geocentric position $r_{9}$ after the diurnal parallax and aberration correction

The corrections for both the diurnal parallax and the diurnal aberration of a star are very small and can be ignored. Then:

(28) $$r_{9}=r_{8}$$

11. Calculate the station center position $r_{10}$ after atmospheric refraction correction

When starlight enters the Earth’s atmosphere, it bends, causing the apparent altitude of celestial bodies to be higher than their true altitude. The effect is greatest when celestial bodies are close to the horizon and zero when they are at the zenith. Its correction model is:

(29) $$\rho =\rho_{0}(1+\alpha A+B)$$

In Equation (29), A represents the constant of temperature change, α is the correction of the constant of temperature change, B is the constant of air pressure change, and $\rho_{0}$ is the difference in atmospheric refraction under standard conditions. Atmospheric refraction can cause intolerable system aberrations that cannot be ignored.

Atmospheric refraction is a local effect that only changes the direction of the target relative to the observation station, without altering the azimuth. Therefore, the correction must be carried out in a reference frame centered on the observation station. When correcting atmospheric refraction, the first step is to convert the geocentric position to the station center position:

(30) $$r_{10}=C\cdot (r_{9}-r_{r})$$

In Equation (30), C represents the rotation matrix from the center of the Earth to the center of the station, and $r_{r}$ is the position of the station. By converting the station center’s rectangular coordinates to spherical coordinates and then performing atmospheric refraction correction, we can obtain:

(31) $$\begin{array}{l} A=\arctan\frac{y_{10}}{x_{10}}\\ h=\arctan\frac{z_{10}}{\sqrt{x_{10}^{2}+y_{10}^{2}}}+\rho \end{array}$$

### 2.3. Simplification of the Time System

In Section 2.2, the approximations and simplifications during the spatial model transformation process were analyzed, and the correction terms less than 0.1″ were ignored. However, the changes in the time system during the calculation process are also very cumbersome. The following is a detailed analysis of the time calculation process involved in Section 2.2. The simplification of the time system for ground arcsecond-level CNS/INS integrated attitude determination is achieved.

The difference between TT and UTC is:

(32) TT−UTC=69.184 s

In the calculation formula of the precession parameter in Equation (13), if UTC is used instead of TT, its influence on the precession matrix can be expressed as:

(33) $$\left\{\begin{array}{c} \delta \zeta_{A}=2306.2181\prime \prime \times \;69.184/(24\;\times \;3600\;\times \;36525)\approx 0\\ \delta \theta_{A}=2004.3109\prime \prime \times \;69.184/(24\;\times \;3600\;\times \;36525)\approx 0\\ \delta Z_{A}=2306.2181\prime \prime \times \;69.184/(24\;\times \;3600\;\times \;36525)\approx 0 \end{array}\right.$$

Similarly, in the calculation formula of the nutation matrix in Equation (18), if UTC is used instead of TT, its influence on the nutation matrix can be expressed as:

(34) $$\left\{\begin{array}{cc} \delta (\Delta \psi ) & \approx A_{1}\sin(N_{51}\delta F_{5})\leq \left|A_{1}N_{51}\delta F\right|\\ & =17.1996\times \;6962890.2665\times 69.184/(24\times 3600\times 36525)\approx 0\\ \delta (\Delta \varepsilon ) & \approx B_{1}\sin(N_{51}\delta F_{5})\leq \left|B_{1}N_{51}\delta F\right|\\ & =9.2052\times 6962890.2665\times 69.184/(24\times 3600\times 36525)\approx 0 \end{array}\right.$$

In the calculation of GMST in Equation (22), if UTC is used instead of TT, it can be expressed as:

(35) δEc=4612.1573966×69.184/(24×3600×36525)≈0

In the calculation of $\theta_{E}$ in Equation (23), if the input time differs from UT1 by 1 second, then $\delta \theta_{E}$ can be expressed as:

(36) $$\delta \theta_{E}=2\pi \times 1.00273781191135448\times 1/(24\times 3600)=15\prime \prime$$

Therefore, in the calculation process of the precession matrix, nutation matrix, and GMST of the CNS/INS integrated navigation, UTC can be directly used to replace TT or TDB. To ensure that the calculation accuracy of $\theta_{E}$ reaches 0.1″, it is necessary to accurately obtain the value of UT1-UTC, which can be obtained from the IERS official website.

### 2.4. Simplification of the Calibration Model

In addition to the transformation from the celestial coordinate system to the Earth coordinate system, to achieve ground-based arcsecond-level astronomical attitude determination results, it is also necessary to obtain the precise coordinates of the star points in the camera coordinate system. By virtue of the feature that the camera position change does not alter the star distribution, the pixel coordinates of the star points are used as observations, and a star calibration method based on the cosine value of the inter-star angular distance as a constraint is adopted to determine the camera’s internal parameters and distortion parameters [28,29,30].

**Figure 2:**
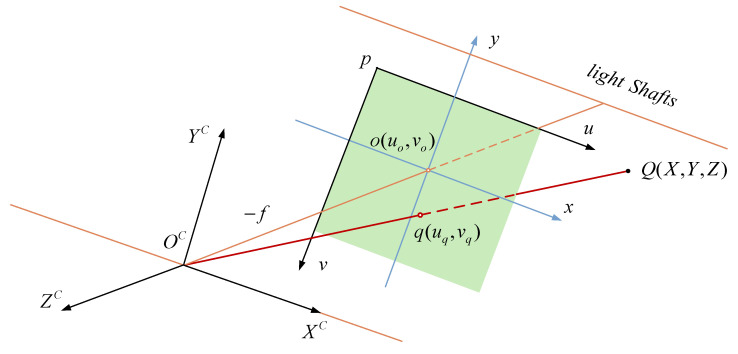
Conversion from pixel coordinates to camera coordinates.

As shown in Figure 2, if the star point Q is imaged at the q point on the image plane, and its coordinate in the pixel coordinate system p−uv is ${\left[u_{q}\;v_{q}\right]}^{T}$, and if the pixel coordinate of the principal point of the image is ${\left[u_{o}\;v_{o}\right]}^{T}$, and the focal length of the camera is f, then the coordinate ${\left[X_{q}\;Y_{q}\;Z_{q}\right]}^{T}$ of q in the camera coordinate system $O^{C}-X^{C}Y^{C}Z^{C}$ we can be expressed by Equation (37):

(37) $${\left[X_{q}\;Y_{q}\;Z_{q}\right]}^{T}={\left[u_{q}-u_{o}\;v_{o}-v_{q}\;-f\right]}^{T}$$

Considering the influence of the distortion [Δu Δv] on the pixel coordinates, Equation (37) can be further expressed as:

(38) $${\left[X_{q}\;Y_{q}\;Z_{q}\right]}^{T}={\left[u_{q}-u_{o}-\Delta u\;v_{o}-v_{q}+\Delta v\;-f\right]}^{T}$$

From Equation (38), it can be known that accurately calibrating the camera’s internal parameters $u_{o}$, $v_{o}$, f and the distortion quantity Δu and Δv is the key to high-precision coordinate transformation. The correction of the star’s pixel coordinate due to distortion can be expressed as:

(39) $$\left\{\begin{array}{c} \Delta u=k_{1}\cdot u\cdot r^{2}+k_{2}\cdot u\cdot r^{4}+k_{3}\cdot u\cdot r^{6}+p_{1}(r^{2}+2u^{2})+2p_{2}uv\\ \Delta v=k_{1}\cdot v\cdot r^{2}+k_{2}\cdot v\cdot r^{4}+k_{3}\cdot v\cdot r^{6}+p_{2}(r^{2}+2v^{2})+2p_{1}uv \end{array}\right.$$

In Equation (39), $u=u_{q}-u_{o}$, $v=v_{o}-v_{q}$, $r=\sqrt{\left(u^{2}+v^{2}\right)}$, and $k_{1}$, $k_{2}$, $k_{3}$, $p_{1}$, $p_{2}$ represent different orders of radial and tangential distortion coefficients.

If the camera field of view is 20°, the ratio of radial distortion of order 2 and 3 and tangential distortion of order 1 and 2 can be represented by $R_{1}$, $R_{2}$, $R_{3}$

(40) $$\left\{\begin{array}{l} R_{1}=\left|\frac{k_{2}}{k_{1}}\right|\cdot {r^{\prime }}^{2}\\ R_{2}=\left|\frac{k_{3}}{k_{1}}\right|\cdot {r^{\prime }}^{4}\\ R_{3}=\frac{\left|p_{1}\right|+\left|p_{2}\right|}{\left|k_{1}\right|\cdot r^{\prime }} \end{array}\right.$$

The coefficients of higher order in Equation (40) are much smaller than those of lower order, $r^{\prime }$ is the normalized radius, let $\left|k_{2}\right|\approx 10^{-2}\left|k_{1}\right|$, $\left|k_{3}\right|\approx 10^{-4}\left|k_{1}\right|$, $\left|p_{1}\right|,\left|p_{2}\right|\approx 10^{-4}\left|k_{1}\right|$, Equation (40) can be expressed as

(41) $$\left\{\begin{array}{c} R_{1}\approx 3.09\times 10^{-4}\\ R_{2}\approx 9.59\times 10^{-6}\\ R_{3}\approx 1.13\times 10^{-3} \end{array}\right.$$

Taking the 20° field of view as an example, the maximum value of is about 0.176, so both $R_{1}$, $R_{2}$, $R_{3}$ are far less than 1. Even though radial distortion of order 1 causes an error of 100 pixels, the effect of distortion of other orders is negligible. Thus, Equation (39) can be expressed as follows.

(42) $$\left\{\begin{array}{c} \Delta u=k_{1}\cdot u\cdot r^{2}\\ \Delta v=k_{1}\cdot v\cdot r^{2} \end{array}\right.$$

Stars can be regarded as ideal point light sources. It is assumed that the interstellar angular distances between stars are equal in both the camera coordinate system and the inertial coordinate system. Using the cosine value of the interstellar angular distance $\theta_{ij}(i=1\ldots n,j=1\ldots n,i\neq j)$, denoted as $\cos(\theta_{ij})$, as the direct observable, the observation equation can be established as shown in Equation (43):

(43) $$\cos(\theta_{ij})=v_{i}^{T}v_{j}=w_{i}^{T}w_{j}$$

In Equation (43), $v_{i}$ and $v_{j}$ respectively represent the unit vectors of the i-th and j-th stars in the navigation coordinate system, which can be calculated from the apparent positions of the stars; $w_{i}$ and $w_{j}$ respectively represent the unit vectors of the i-th and j-th stars in the camera coordinate system.

Let:

(44) $$\begin{array}{c} D_{i}=\sqrt{{\left((u_{i}-u_{o})/f-\delta u_{i}\right)}^{2}+{\left((v_{o}-v_{i})/f+\delta v_{i}\right)}^{2}+1}\\ D_{j}=\sqrt{{\left((u_{j}-u_{o})/f-\delta u_{j}\right)}^{2}+{\left((v_{o}-v_{j})/f+\delta v_{j}\right)}^{2}+1}\\ N_{ij}=((u_{i}-u_{o})/f-\delta u_{i})((u_{j}-u_{o})/f-\delta u_{j})+((v_{o}-v_{i})/f+\delta v_{i})((v_{o}-v_{j})/f+\delta v_{j})+1\\ g_{ij}=w_{i}^{T}w_{j}=\frac{N_{ij}}{D_{i}D_{j}} \end{array}$$

The linearized error equation can be expressed as:

(45) $$\begin{array}{ll} R_{ij} & =w_{i}^{T}w_{j}-v_{i}^{T}v_{j}\\ & =\;g_{ij}-v_{i}^{T}v_{j}\\ & =\left[\begin{array}{cccc} \frac{\partial g_{12}}{\partial u_{o}} & \frac{\partial g_{12}}{\partial v_{o}} & \frac{\partial g_{12}}{\partial f} & \frac{\partial g_{12}}{\partial k_{1}}\\ \frac{\partial g_{13}}{\partial u_{o}} & \frac{\partial g_{13}}{\partial v_{o}} & \frac{\partial g_{13}}{\partial f} & \frac{\partial g_{13}}{\partial k_{1}}\\ \vdots & \vdots & \vdots & \vdots \\ \frac{\partial g_{ij}}{\partial u_{o}} & \frac{\partial g_{ij}}{\partial v_{o}} & \frac{\partial g_{ij}}{\partial f} & \frac{\partial g_{ij}}{\partial k_{1}} \end{array}\right]\left[\begin{array}{c} \Delta u_{o}\\ \Delta v_{o}\\ \Delta f\\ \Delta k_{1} \end{array}\right]-\left[\begin{array}{c} v_{1}^{T}v_{2}-\frac{N_{12}}{D_{1}D_{2}}\\ v_{1}^{T}v_{3}-\frac{N_{13}}{D_{1}D_{3}}\\ \vdots \\ v_{i}^{T}v_{j}-\frac{N_{ij}}{D_{i}D_{j}} \end{array}\right] \end{array}$$

By using the least squares iterative method for solution, the internal orientation elements and distortion parameters of the camera can be calculated simultaneously.

## 3. Experimental Verification and Analysis

To verify that the simplification carried out can meet the ground arcsecond-level attitude determination requirements, this section first introduces the sensor parameters of the experimental platform used in the experiment; then analyzes the impact of each simplification term on attitude determination and the applicability of the star calibration method adopted; finally, based on the rigorous model and the simplified model, the carrier attitude is calculated, and the validity of the simplified model proposed in this paper is verified through the consistency of the attitude determination results of the two models.

### 3.1. Experimental Platform

**Figure 3:**
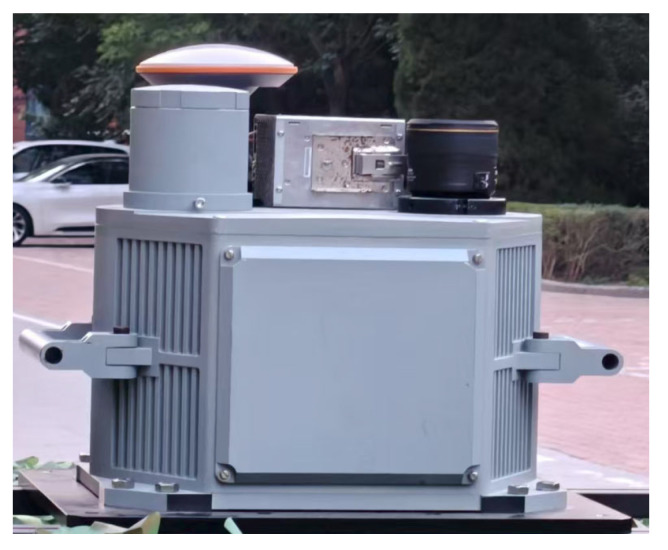
CNS/INS integrated navigation experiment platform.

To verify the feasibility of the method proposed in this paper, a combined navigation experiment was conducted. The experiment was carried out in the High-tech Zone of Zhengzhou City in March 2025. The experimental platform is shown in Figure 3. The platform was equipped with a CNS/INS combined navigation device. The specific parameters of the astronomical sensor are given in Table 2. The star sensor model adopted was QHY42PR, equipped with a Nikon lens, with a field of view of approximately 20°. The pixel size of the adopted COMS sensor is 11 × 11, and the resolution was 2048 pixels × 2048 pixels. The pinhole imaging model was used as the projection model, and one image was collected every 5 s.

### 3.2. Influence of Annual Parallax and Light Deflection

**Figure 4:**
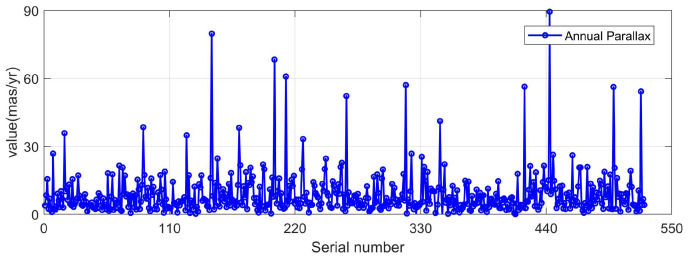
Annual parallax of stars.

**Figure 5:**
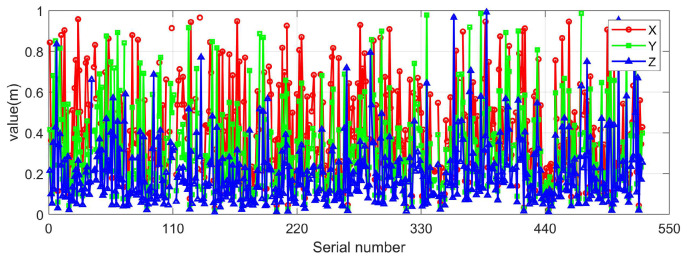
Ignore the errors in the heliocentric position in the X, Y, and Z directions caused by light deflection.

To verify the influence of ignoring the annual parallax correction and the light deflection correction on the heliocentric position, in the designed navigation star library, the star points in the field of view were determined according to the direction of the collimation axis. The heliocentric position errors of these star points with and without the light deflection correction were given, respectively, as shown in Figure 4 and Figure 5. It can be seen from the figures that the annual parallax of the stars in the current star map field of view is relatively small, with the maximum not exceeding 90 milliarcseconds per year, less than 0.1 arcsecond. The maximum error in the three directions of the heliocentric position does not exceed 1 m. Therefore, the influence of the two corrections on the heliocentric position can be ignored.

### 3.3. Impact of Simplified Calculation of Nutation and Precession and GAST on Combined Attitude Determination

**Figure 6:**
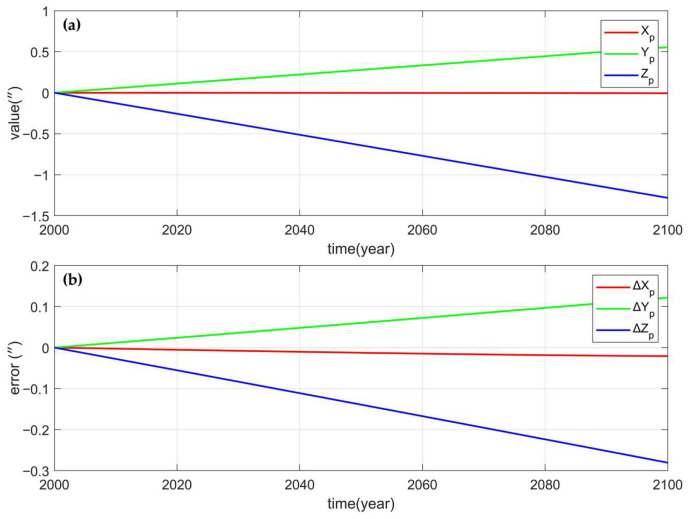
(**a**) is the precession Euler Angle ($X_{p}$, $Y_{p}$, $Z_{p}$) calculated by Novas; (**b**) is the error of the precession Euler Angle calculated by the simplified model (${∆X}_{p}$, $∆Y_{p}$, ${∆Z}_{p}$).

**Figure 7:**
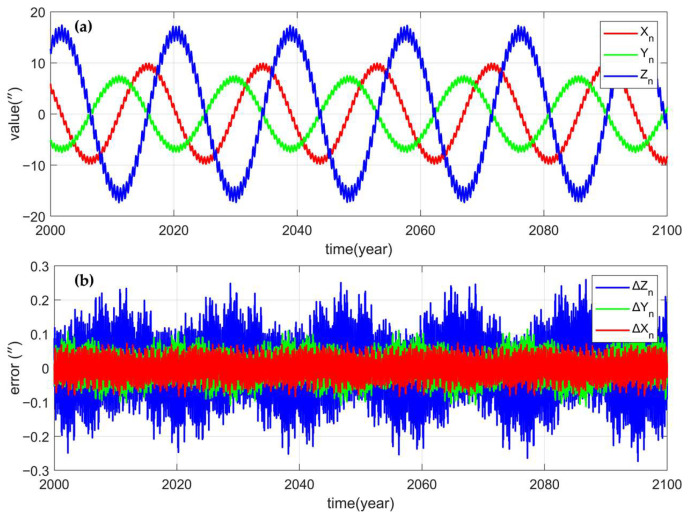
(**a**) is the perturbation Euler angles ($X_{n}$, $Y_{n}$, $Z_{n}$) calculated by Novas; (**b**) is the errors of the perturbation Euler angles calculated by the simplified model (${∆X}_{n}$, $∆Y_{n}$, ${∆Z}_{n}$).

**Figure 8:**
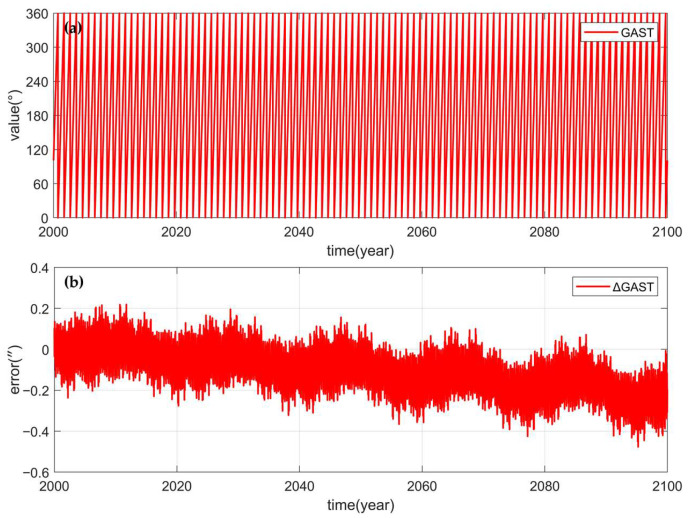
(**a**) is the GAST Angle calculated by Novas; (**b**) is the GAST Angle error calculated by the simplified model (∆GAST).

To verify whether the simplified calculation of precession, nutation and GAST meets the accuracy requirements of ground CNS/INS integrated attitude determination, the precession, nutation Euler angles and GAST angles from the J2000 epoch to the year 2100 were calculated for a total of 100 years, and the results were compared with those calculated by Novas. Figure 6a, Figure 7a and Figure 8a show the precession, perturbation Euler Angle, and GAST Angle errors of the Novas output, and Figure 6b, Figure 7b and Figure 8b show the differences between the calculated values of the simplified model proposed in this paper and the Novas output values. Comparative analysis reveals that, compared with Novas, the errors of the precession, nutation Euler angles, and GAST angles calculated using the simplified model are all within 0.5”, and before 2050, the errors are all within 0.2”. This indicates that the simplified model can achieve high-precision conversion between the celestial coordinate system and the Earth coordinate system.

### 3.4. Impact of Simplified Calculation on Calibration Model 

**Figure 9:**
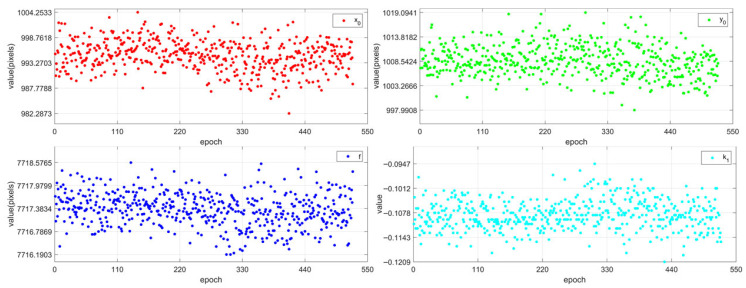
Results of calibration for multiple star map camera parameters.

**Figure 10:**
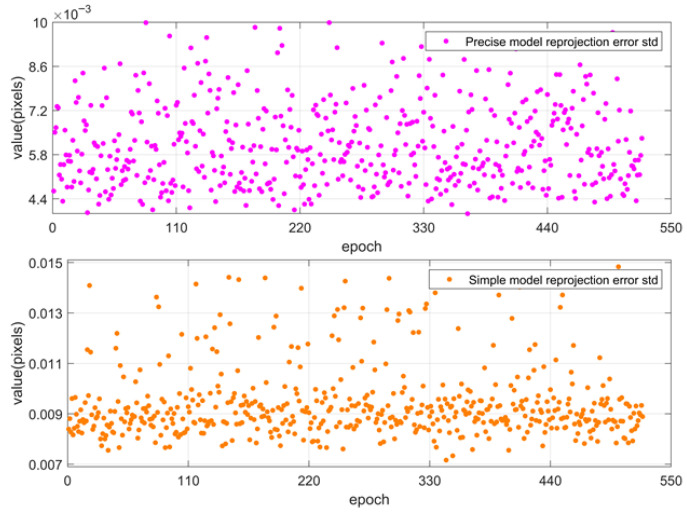
Reprojection errors of the precise model parameters and those of the simplified model parameters.

The simplified model was used to calibrate the camera parameters of the successfully matched multiple star maps. Figure 9 shows the calibration parameters obtained, and Table 3 presents the mean and standard deviation of the parameters $u_{o}$, $v_{o}$, *f*, and $k_{1}$ of multiple star maps. The calibration parameters obtained from each star map are relatively stable. The variation range of the focal length *f* is from 7716.1903 to 7718.5765 pixels; the variation range of the principal point $u_{o}$ is from 982.2873 to 1004.2533 pixels; the variation range of the principal point $v_{o}$ is from 997.9908 to 1019.0941 pixels; and the variation range of the first-order radial distortion $k_{1}$ is from −0.1209 to −0.0947. Figure 10 shows the reprojection errors of the precise and simplified models of the camera parameters of multiple star maps. The calibration accuracy of each star map meets the requirements, with the maximum absolute mean value of the reprojection error of the precise model not exceeding 0.01 pixels and that of the simplified model not exceeding 0.015 pixels. The corresponding maximum attitude system difference is approximately 0.3″. The parameters calculated by the adopted simplified calibration model meet the requirements of ground-level arcsecond-level attitude determination. 

### 3.5. Comparative Analysis of Attitude Determination Results

**Figure 11:**
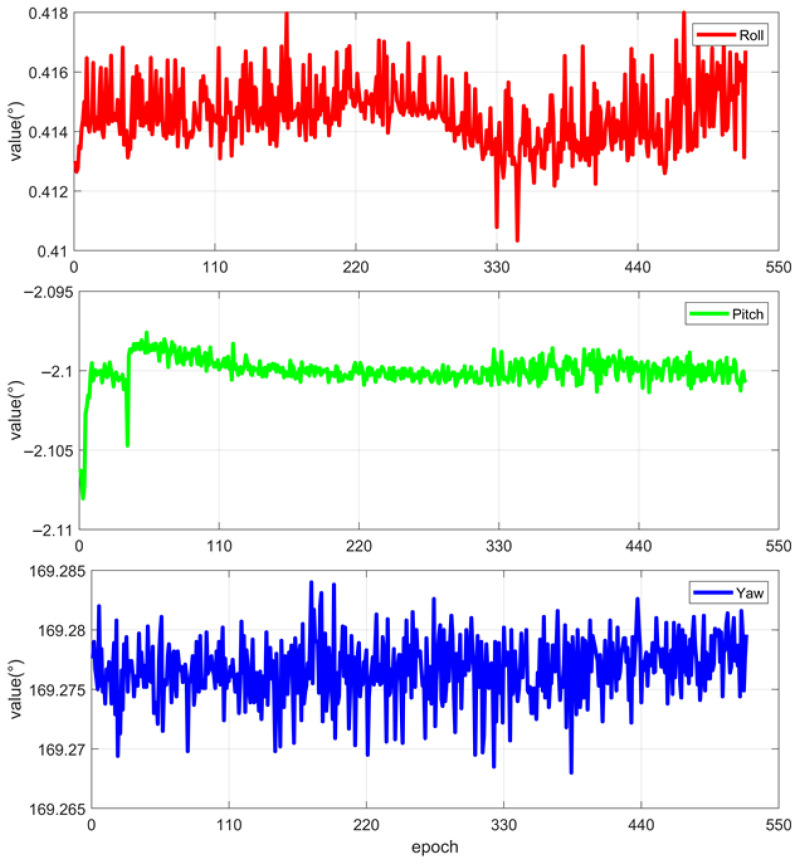
Results of precise model solution.

**Figure 12:**
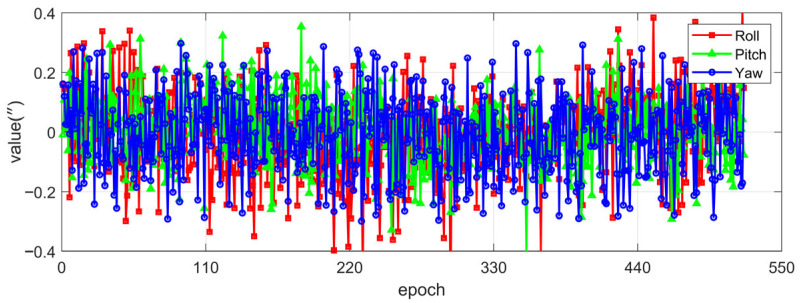
The difference between the calculated pose of the simplified model and that of the precise model.

To further verify the influence of the simplified model on the actual attitude determination, the measured data were processed for attitude calculation using both the simplified model and the precise model. Figure 11 shows the attitude results calculated by the precise model. The initial fluctuation in the pitch direction is due to the person getting on and off the vehicle. Table 4 presents the standard deviations (STD) in the three directions. The STD of the horizontal attitude is less than 5″, while that of the heading attitude is 9.30″, which is attributed to the poor observability of the heading in static observation. Figure 12 shows the attitude errors between the simplified model and the precise model. By comparison, it can be seen that the attitude error calculation results of the three axes of the proposed simplified model in this paper are all better than 0.4 arcseconds, meeting the attitude determination accuracy requirements of the SINS/CNS combination.

## 4. Discussion

Although this paper has verified the method under ground static conditions, the principles of model construction and simplification are not limited to this scenario. For airborne, shipborne, or spaceborne platforms, if the attitude accuracy indicators required by the mission are of the same order of magnitude as those in this study, and the observation conditions allow the avoidance of strongly influential factors, the same simplification strategy is theoretically still applicable. Future work will focus on conducting experimental verification in dynamic and space environments to further quantify the applicability boundaries of this method in different application scenarios.

Due to the advantages of this simplified model in terms of computational load, it can be directly deployed to the on-board processing unit of star sensors, enabling real-time processing of edge computing and data fusion. Furthermore, the stellar self-calibration technology supports adaptation to environmental changes, realizing the “immediate inspection and immediate use” function without dedicated calibration, and exhibits application potential for intelligent sensor systems.

## 5. Conclusions

This paper presents a simplified spatiotemporal model for CNS/INS integrated attitude determination, which can help ordinary navigation professionals get started quickly. According to the ground arcsecond-level CNS/INS integrated navigation and attitude determination requirements, the factors that need to be considered in the spatio-temporal transformation process are analyzed one by one first, and the influencing factors with a contribution less than 0.1″ are discarded; then a camera internal parameter calibration method with stars as high-precision references is adopted; finally, the effectiveness of the proposed method in this paper is verified through actual measurement experiments. The main conclusions are as follows:

1. During the transformation between the celestial coordinate system and the Earth coordinate system, the annual parallax and the light deflection can be disregarded. The calculation of GAST requires a relatively precise UT1-UTC, while for other parameters, UTC can be used as a substitute. Under the ground-based attitude determination requirement of arcsecond level, the calibration model of small field-of-view star sensors can adopt only the first-order radial distortion. The difference in attitude accuracy between the simplified model solution and the precise model solution is less than 0.4 arcseconds.
2. The star calibration method adopted in this paper can reduce the parameter deviation between the laboratory and the actual environment, achieving the effect of on-demand calibration. It has high parameter solution accuracy and strong robustness, and can meet the requirements of the CNS/INS integrated attitude determination at the arcsecond level.

## Figures and Tables

**Table 1:** IAU1980 nutation series listed according to abs ($A_{i}$).

Parameter	Argument Coefficients					Longitude Nutation Coefficient/(″)		Obliquity Nutation Coefficient/(″)	
i	$N_{1t}$	$N_{2t}$	$N_{3t}$	$N_{4t}$	$N_{5t}$	$A_{i}$	$A_{i}^{\prime }$	$B_{i}$	$B_{i}^{\prime }$
1	0	0	0	0	1	−17.1996	−0.001742	9.2052	0.00089
2	0	0	2	−2	2	−1.3187	−0.00016	0.5736	−0.00031
3	0	0	2	0	2	−0.2774	−0.00002	0.0977	−0.00005
4	0	0	0	0	2	0.2062	0.00002	−0.0895	0.00005
5	0	1	0	0	0	0.1426	−0.00034	0.0054	−0.00001
6	1	0	0	0	0	0.0712	0.00001	−0.0007	0.0

**Table 2:** Navigation performance of the sensors.

Sensor	Parameter	Values
CNS	Field of view	20°
	Pixel size	11 × 11
	Image sensor size	2048 pixels × 2048 pixels
	Observation time for single star map	5 s

**Table 3:** The parameter calibration results of multiple star map cameras.

	*f*/Pixels	$u_{0}$/Pixels	$v_{0}$/Pixels	$k_{1}$
Mean	7717.34	994.90	1008.64	−0.108
Std	0.43	3.26	3.37	0.004

**Table 4:** STD in three directions.

	Roll/″	Pitch/″	Yaw/″
Std	3.39	3.83	9.30

## Data Availability

The dataset used in this paper can be obtained by reasonable request to the corresponding author.

## References

[1] Jiang H., Zhang Y., Zhao Y. (2024). A Novel SINS/CNS Integrated Navigation Scheme Using Rodrigues Parameters. IEEE Sens. J..

[2] Shi B., Wang M., Wang Y., Bai Y., Lin K., Yang F. (2021). Effect Analysis of GNSS/INS Processing Strategy for Sufficient Utilization of Urban Environment Observations. Sensors.

[3] Mu R., Sun H., Li Y., Cui N. (2020). INS/CNS Deeply Integrated Navigation Method of Near Space Vehicles. Sensors.

[4] Wang Q., Cui X., Li Y., Ye F. (2017). Performance Enhancement of a USV INS/CNS/DVL Integration Navigation System Based on an Adaptive Information Sharing Factor Federated Filter. Sensors.

[5] Xu S., Zhou H., Wang J., He Z., Wang D. (2019). SINS/CNS/GNSS Integrated Navigation Based on an Improved Federated Sage–Husa Adaptive Filter. Sensors.

[6] Tian Z., Cheng Y., Yao S., Li Z. (2024). An Adaptive INS/CNS/SMN Integrated Navigation Algorithm in Sea Area. Remote Sens..

[7] Zhao Y., Yan G., Qin Y., Fu Q. (2020). Information Fusion Based on Complementary Filter for SINS/CNS/GPS Integrated Navigation System of Aerospace Plane. Sensors.

[8] Atkinson D., Agnew J., Miller M. (1993). The B-2 Navigation System. Proceedings of the Proceedings of the IEEE 1993 National Aerospace and Electronics Conference-NAECON 1993.

[9] Cheng F. (2019). Algorithm Design and Implementation of CNS/GNSS/INS High Precision Ship-borne Real-time Attitude Determination System. Bull. Surv. Mapp..

[10] Li Z., Zhan Y., Du H., Shen Y., Chen S., Zhang C. (2024). Robust Adaptive SINS/CNS Integrated Navigation Algorithm for Mars Rovers with Experimental Verification. Measurement.

[11] Liu J. (2013). A Research on the New Astronomical Reference System. Acta Astron. Sin..

[12] Petit G., Luzum B. IERS Technical Note. No. 36. https://iers-conventions.obspm.fr/content/tn36.pdf.

[13] Bennett G.G. (1979). General Conventions and Solutions-Their Use in Celestial Navigation. Navigation.

[14] Yan G., Dai C., Chen R. (2022). Space-time coordinate transformation and its error analysis for INS/CNS integrated navigation. Zhongguo Guanxing Jishu Xuebao.

[15] Abdel-Aziz Y.I., Karara H.M. (2015). Direct Linear Transformation from Comparator Coordinates into Object Space Coordinates in Close-Range Photogrammetry. Photogramm. Eng. Remote Sens..

[16] Zhang Z. (2000). A Flexible New Technique for Camera Calibration. IEEE Trans. Pattern Anal. Mach. Intell..

[17] Ahmed M., Farag A. (2005). Nonmetric Calibration of Camera Lens Distortion: Differential Methods and Robust Estimation. IEEE Trans. Image Process..

[18] Hassanpour R., Atalay V. (2004). Camera Auto-Calibration Using a Sequence of 2D Images with Small Rotations. Pattern Recognit. Lett..

[19] Frahm, Koch (2003). Camera Calibration with Known Rotation. Proceedings of the Ninth IEEE International Conference on Computer Vision.

[20] Vasconcelos F., Barreto J.P., Boyer E. (2018). Automatic Camera Calibration Using Multiple Sets of Pairwise Correspondences. IEEE Trans. Pattern Anal. Mach. Intell..

[21] Kim J.O., Lee D. (2021). Mathematical Model for a Calibration of Multiple-View Thermal Camera. Proceedings of the 2021 International Conference on Electronics, Information, and Communication (ICEIC).

[22] Hartley R.I. (1994). Projective Reconstruction and Invariants from Multiple Images. IEEE Trans. Pattern Anal. Mach. Intell..

[23] Klioner S.A. (2007). Relativistic Scaling of Astronomical Quantities and the System of Astronomical Units. Astron. Astrophys..

[24] Zhan Y., Zhang C., Zhou W., Chen S., Li X. (2025). Theory and Precision Analysis of Astrogeodetic Vertical Deflection Determination Using an Imaging Total Station. J. Geod..

[25] Shi C., Zhang C., Du L., Li J., Ye K., Zhang W., Chen C., Li C., Ma L., Lin H. (2020). Automatic Astronomical Survey Method Based on Video Measurement Robot. J. Surv. Eng..

[26] Li C. (2013). Research on Marine Celestial Navigation Based on Fisheye Camera. Bull. Surv. Mapp..

[27] Cao N. (2014). Research on the Star’s Apparent Position Algorithm Applying to Geodetic Astronomical Surveying. Master’s Thesis.

[28] Pal M., Bhat M.S. (2015). Autonomous Star Camera Calibration and Spacecraft Attitude Determination. J. Intell. Robot. Syst..

[29] Pal M., Bhat M.S. (2009). Star Camera Calibration Combined with Independent Spacecraft Attitude Determination. Proceedings of the 2009 American Control Conference.

[30] Pal M., Bhat M.S. (2013). Star Sensor Based Spacecraft Angular Rate Estimation Independent of Attitude Determination. Proceedings of the 2013 IEEE International Conference on Control Applications (CCA).

